# Body Gaze as a Marker of Sexual Objectification: A New Scale for Pervasive Gaze and Gaze Provocation Behaviors in Heterosexual Women and Men

**DOI:** 10.1007/s10508-022-02290-y

**Published:** 2022-03-29

**Authors:** Ross C. Hollett, Shane L. Rogers, Prudence Florido, Belinda Mosdell

**Affiliations:** Cognition Research Group, Psychology and Criminology, Edith Cowan University, 270 Joondalup Drive, Joondalup, WA 6027 Australia

**Keywords:** Sexual objectification, Body, Gaze, Eye tracking

## Abstract

Body gaze behavior is assumed to be a key feature of sexual objectification. However, there are few self-report gaze measures available and none capturing behavior which seeks to invite body gaze from others. Across two studies, we used existing self-report instruments and measurement of eye movements to validate a new self-report scale to measure pervasive body gaze behavior and body gaze provocation behavior in heterosexual women and men. In Study 1, participants (*N* = 1021) completed a survey with newly created items related to pervasive body gaze and body gaze provocation behavior. Participants also completed preexisting measures of body attitudes, sexual assault attitudes, pornography use, and relationship status. Exploratory and confirmatory factor analyses across independent samples suggested a 12-item scale for men and women to separately measure pervasive body gaze (5 items) and body gaze provocation (7 items) toward the opposite sex. The two scales yielded excellent internal consistency estimates (.86–.89) and promising convergent validity via positive correlations with body and sexual attitudes. In Study 2, a subsample (*N* = 167) of participants from Study 1 completed an eye-tracking task to capture their gaze behavior toward matched images of partially and fully dressed female and male subjects. Men exhibited body-biased gaze behavior toward all the female imagery, whereas women exhibited head-biased gaze behavior toward fully clothed male imagery. Importantly, self-reported body gaze correlated positively with some aspects of objectively measured body gaze behavior. Both scales showed good test–retest reliability and were positively correlated with sexual assault attitudes.

**Supplementary Information:**

The online version contains supplementary material available at 10.1007/s10508-022-02290-y.

## Introduction

Body-biased gaze has long been recognized as an important feature of sexual objectification (Bartky, 1990; Fredrickson & Roberts, 1997). Body-biased gaze is the preferential visual attention toward body parts (compared to the face), often characterized as undesirable, a possible precursor to social deviance (e.g., sexual harassment or assault), and typically observed in men toward women (Bareket et al., 2018; Gervais et al., 2013, 2018; Hollett et al., 2019; Kozee et al., 2007). Only recently has the association between self-reported objectifying attitudes and eye movements been explored, with preliminary evidence suggesting that body-biased gaze is one behavioral manifestation of sexual objectification attitudes (Bareket et al., 2018; Hollett et al., 2019). Therefore, the propensity to exhibit body-biased gaze may be a valuable marker for sexual attitudes, intentions, and behaviors in both women and men. Furthermore, we argue that the propensity to invite body gaze from others might also be a marker for sexual attitudes, intentions, and behaviors in both women and men. However, few measurement options exist for gaze behaviors toward the bodies of others, that we term pervasive body gaze, and behaviors which encourage others to gaze at the self, that we term body gaze provocation. Consequently, we aimed to develop and validate a new self-report instrument to measure attitudes and behaviors related to pervasive body gaze and body gaze provocation to extend our understanding of sexual objectification.

### Sexual Objectification and Gaze Behavior

Sexual objectification can be understood as the separation of a person from their body or body parts and prioritizing these features when determining their worth, usually at the expense of their emotional, social, or intellectual worth (Bareket et al., 2018; Bartky, 1990; Holland & Haslam, 2013). This understanding has since been extended to involve broader underlying mechanisms such as decreased moral concern and competency (Fasoli et al., 2018; Loughnan et al., 2010; Smith et al., 2018), dehumanization (Rudman & Mescher, 2012), and relaxed attitudes toward the sexual assault of women (Bernard et al., 2015; Loughnan et al., 2013; Vega & Malamuth, 2007). These mechanisms can be used to explain subtle indicators of sexual objectification, such as measurable cognitive biases (e.g., Rudman & Mescher, 2012; Yao et al., 2010), through to more extreme intentions or behaviors, such as the perpetration of sexual assault (Gervais et al., 2018; Kozee et al., 2007).

The implications of these objectifying attitudes and behaviors are immense because they are critical for understanding how women, in particular, can become undervalued and mistreated. However, a framework for explaining and predicting sexual objectification needs to also acknowledge that women can sexually objectify men through similar behaviors (Bernard et al., 2018). It is important to recognize that the underlying mechanisms driving these behaviors might be fundamentally different for women and men. For instance, gender variations in dehumanization, appraisals of body shape, and strategies for influencing social power may differentially explain similar manifestations of sexually objectifying behaviors (Gervais et al., 2020; Oehlhof et al., 2009; Vaes et al., 2011). Developing instruments to compare across heterosexual women and men is imperative for understanding the mechanisms underlying sexually objectifying attitudes, intentions, and behavior.

One sexually objectifying behavior exhibited by heterosexual men and women is body-biased gaze (Bernard et al., 2018; Hollett et al., 2019; Lykins et al., 2008), although it is most commonly observed in men toward women (e.g., Bareket et al., 2018; Fredrickson & Roberts, 1997). Receiving body-biased gaze is potentially harmful, particularly to women, with links to decreased cognitive performance, increased self-objectification, and unwanted sexual advances (Calogero, 2004; Gervais et al., 2011; Holland et al., 2017; Kozee et al., 2007). Importantly, body gaze behavior can be influenced by the appearance of the recipient (Dixson et al., 2009; Gervais et al., 2013; Hollett et al., 2019; Smith et al., 2018), suggesting that the intentions, attitudes, and behaviors of gaze recipients need to be measured when describing heterosexual female and male gaze patterns.

Depictions of women in mainstream media provide useful examples of a persistent provocation phenomena. Advertising, music videos, video games, and pornography employ common strategies for manipulating the visual attention of consumers (e.g., Behm-Morawitz, 2017; Burgess et al., 2007; Downs & Smith, 2010; Jansz & Martis, 2007; Karsay & Matthes, 2020; Karsay et al., 2018; McKenzie-Mohr & Zanna, 1990; Stankiewicz & Rosselli, 2008; Tylka & Kroon Van Diest, 2014). These strategies involve tight clothing, revealing of skin, and a narrow focus on bodily features, such as legs, chests/breasts, and buttocks. Because these observations are largely derived from media analyses examining women, further research is also needed to better understand the impact of men acting and dressing in provocative ways.

Sexualized depictions of women and men are arguably capable of facilitating body-biased patterns of behavior through at least two mechanisms. Firstly, repeated exposure to sexualized depictions of people may prime consumers to habitually adopt body-biased gaze behavior, which may then extend to real people (Hollett et al., 2019; Karsay et al., 2018; Tyler et al., 2017). Secondly, sexualized depictions of women and men may normalize styles of physical presentation and encourage people to dress and behave in ways which draws attention to their bodies (Calogero & Tylka, 2014; Smolak et al., 2014). Through deliberate adoption or implicit internalization of these norms, adopting clothing and behavior which invites the gaze of others toward one’s own body, particularly from the opposite sex, can be described as provocative. While intentional or unintentional provocation of body gaze is important for influencing human gaze behavior, it is still poorly understood, possibly due to a lack of measurement options for estimating the propensity of women and men to provoke body gaze. Therefore, any measurement agenda for furthering the understanding of sexually objectifying gaze needs to account for gaze toward others and gaze toward the self.

### Toward a More Balanced Understanding of Sexually Objectifying Gaze

To advance our understanding of gaze behavior as a feature of sexual objectification, it is important to identify the factors which facilitate this behavior. One factor involves efforts to draw gaze toward one’s own body, or lack of effort to deter gaze from one’s body. That is, the appearance of the gaze recipient can increase the likelihood that they will receive body gaze from others (Dixson et al., 2009; Gervais et al., 2013; Hewig et al., 2008; Hollett et al., 2019; Lykins et al., 2008). Importantly, we recognize that implicit adoption of sociocultural norms and contextual demands regarding appearance and behavior might contribute to whether body gaze provocation is intentional or incidental. That is, while self-reported measures of body gaze provocation would most likely capture deliberate efforts, responses may also plausibly reflect incidental body gaze provocation.

Similar to body gaze provocation, the tendency to gaze at the bodies of others can involve deliberate effort as well as lack of effort to inhibit gaze behavior. While self-reported measures of body gaze are best capable of capturing deliberate efforts, responses may also plausibly reflect incidental or automatic engagement in body gaze. However, incidental and automatic gaze are best captured using eye tracking technology (Ricciardelli et al., 2013). While the measurement of body gaze continues to be a valuable research objective, few studies have attempted to validate instruments to capture this behavior. This is surprising given how widely sexually objectifying gaze has been described in the extant literature (e.g., Bareket et al., 2018; Calogero, 2004; Fredrickson & Roberts, 1997; Gervais et al., 2011, 2013; Karsay et al., 2018; Loughnan & Pacilli, 2014). To determine an appropriate direction for body gaze measurement, it is useful to summarize and critique currently available methods for measuring body gaze.

### Measurement of Gaze Behavior Toward Men and Women

Eye tracking is the most direct method for capturing body gaze behavior, because it can accurately measure habitual, spontaneous, as well as intentional and effortful gaze behavior (e.g., Ricciardelli et al., 2013). Several prior studies have used eye tracking technology to characterize female and male gaze behavior. Specifically, women and men have been shown to exhibit similar head-biased gaze behavior when presented with images of non-sexualized women and men (Hewig et al., 2008; Hollett et al., 2019; Nummenmaa et al., 2012). However, using more appealing imagery reveals gender differences. In particular, men show stronger body gaze behavior than women toward increasingly sexualized, idealized, or attractive female subjects (Gervais et al., 2013; Lykins et al., 2008; Nummenmaa et al., 2012). Although there is also evidence that women engage in body-biased gaze behavior toward sexualized female subjects (Hollett et al., 2019; Lykins et al., 2008; Nummenmaa et al., 2012), fewer studies have examined gaze behavior toward sexualized men (Bernard et al., 2018; Lykins et al., 2008; Nummenmaa et al., 2012) and suggest that both women and men engage in body-biased gaze behavior toward nude, eroticized, or idealized men, with some evidence of stronger effects in heterosexual women.

Importantly though, there is a strong support for face gaze as a more naturalistic pattern of social attention because it is imperative for detecting emotional and dispositional signals from a person (Castelhano et al., 2007; Emery, 2000; Foulsham et al., 2010; Gobel et al., 2015). These assumptions are supported by several studies showing that the face attracts more visual attention than the body in non-sexualized subjects (Hewig et al., 2008; Hollett et al., 2019; Nummenmaa et al., 2012). Therefore, when body gaze consistently outweighs facial gaze, we might assume it is socially maladaptive or sexually objectifying. Indeed, recent research has begun supporting the utility of body-biased gaze behavior as a marker for sexual objectification (Bareket et al., 2018; Hollett et al., 2019; Riemer et al., 2018). Specifically, gaze behavior in men toward female subjects has been linked to sexually objectifying attitudes toward women and rape myth acceptance attitudes (Bareket et al., 2018; Sussenbach et al., 2015).

Given that very few studies have examined gaze behavior of women and men toward both male and female subjects (Hollett et al., 2019; Lykins et al., 2008; Nummenmaa et al., 2012) or examined associations between gaze and sexually objectifying attitudes (Bareket et al., 2018; Hollett et al., 2019), our understanding of heterosexual gaze behavior is still limited. While eye tracking technology is an important tool for extending our knowledge of gaze behavior and sexual intentions, attitudes, and behaviors, it is not accessible to all sexual objectification researchers. Therefore, developing self-report instruments with some utility for estimating gaze behaviors is a valuable objective.

It would be overambitious to attempt to develop a self-report instrument which simulates or substitutes an eye-tracking procedure. However, there remains utility in a self-report inventory to capture body-biased gaze if it serves as a valid marker for broader sexual objectification attitudes and behaviors. To our knowledge, one self-reported body-biased gaze measure currently exists. In their adaptation of the victim-orientated interpersonal sexual objectification scale (ISOS; Kozee et al., 2007), Gervais et al. (2018) modified the items to instead reflect the perpetration of sexual objectification (ISOS-P) which included a 6-item subscale measuring the frequency that a respondent recalls “staring,” “evaluating,” “leering,” or “gazing” at another person’s body or body parts. This subscale yielded good internal consistency in men (.86) and women (.80), but the Gervais et al. did not report subscale correlations with convergent measures, so it is unclear how self-reported body gaze performs as a marker for other body and gender attitudes.

We also note that the ISOS-P has several limitations. Firstly, most of the items are repetitive, using synonyms (e.g., staring, gazing) rather than attempting to more comprehensively capture the breadth of behaviors that reflect the construct of interest. Secondly, two of their body gaze items (2 and 10) had relatively low loadings (.13–.35) when subject to confirmatory factor analyses (particularly for women). Finally, given that their items were adapted from an already distilled scale that was designed to measure recipient perceptions of body gaze, rather than selected from a large, generated list, it may lack theoretical and practical utility. Sexual objectification has been empirically and theoretically extended in the 10 years following the publication of the Kozee et al. (2007) scale, so it is appropriate to generate new items for the purpose of scale development. In particular, we aimed to develop a body gaze scale referring to broader attitudes and behaviors with pervasive attributes (e.g., effortful and uninhibited) to reflect the deleterious manner in which sexually objectifying gaze is often described in the literature (e.g., Bareket et al., 2018; Fredrickson & Roberts, 1997; Gervais et al., 2013; Holland et al., 2017; Miles-McLean et al., 2014). One possible advantage of a scale which achieves this aim is an increased likelihood that it will overlap with other known, and arguably maladaptive, components of sexual objectification, such as decreased moral concern for women, dehumanization, as well as relaxed sexual assault attitudes and behaviors (Bernard et al., 2015; Loughnan et al., 2010, 2013; Rudman & Mescher, 2012).

Factorial analyses by Gervais et al. (2018) and Kozee et al. (2007) have provided valuable evidence that engaging in, and receiving, body gaze are key features of sexual objectification. However, to our knowledge, these prior instruments have not been validated against eye tracking or subject to test–retest reliability. Furthermore, while the Kozee et al. (2007) scale asks participants to recollect the frequency of receiving body gaze, it does not measure their enjoyment, tolerance, or efforts to invite this behavior from others. That is, the intentions and preferences of the gaze recipient are unclear. By contrast, the Enjoyment of Sexualization Scale for women (Liss et al., 2011) does partly measure women’s enjoyment of being gazed at by men, but does not capture their behavioral efforts or intentions to draw this gaze. While body gaze provocation could be one manifestation of “self-objectification” (Moradi & Varnes, 2017), people who internalize self-objectifying attitudes may not want to promote their bodies to others, due to heightened body shame and anxiety (Moradi, 2010; Moradi & Huang, 2008). However, we still expect that body gaze provocation behaviors may correlate with self-objectifying attitudes.

### The Present Study

Given that the value of psychological constructs rest in the quality of their measurement, we designed a study to evaluate the validity and reliability of an instrument for estimating *pervasive* body gaze tendencies toward others, and *provocative* tendencies which invites body gaze from others upon the self. As both sets of behavioral tendencies are considered outcomes of the same socio-cognitive process involving a propensity to view a person’s body as a valuable consumable entity (Fredrickson & Roberts, 1997; Loughnan & Pacilli, 2014), we treated them as subfactors in our psychometric analyses. That is, we assumed that the process of sexual objectification manifests in the adoption of a set of body-biased behaviors involving both the self and others, and we expected to produce evidence to support this assumption (acceptable model fit and moderate or high positive intercorrelations). Our multi-study design utilized an online administration of scale items to be factor analyzed, followed by laboratory sessions to measure gaze behavior.

In Study 1, we administered the items designed to capture pervasive and provocative body gaze tendencies alongside several convergent measures. Consistent with recent psychometric research (Gervais et al., 2018), self-objectification (Moradi & Varnes, 2017), and eye-tracking research (Hollett et al., 2019), we included validated scales for estimating attitudes toward one’s own body (e.g., shame, monitoring, appearance self-esteem), interpersonal sexual objectification experiences, as well as the sexual assault of women (victim and perpetrator blame). Because pornography is largely designed to prime body gaze behavior (Klaassen & Peter, 2015), we also measured pornography use. We also assumed that promoting one’s body to others might be a strategy for securing sexual partners and, given that number of sexual partners is positively associated with sensation seeking (Charnigo et al., 2013), we measured sensation seeking to assist in the validation of body gaze provocation. The Enjoyment of Sexualization Scale (Liss et al., 2011), validated for women, was also included in Study 1 and has theoretical overlap with our conceptualization of body gaze provocation. Lastly, in Study 1, we measured relationship status to explore the possibility that single participants might be more likely to engage in pervasive body gaze and body gaze provocation compared to partnered participants. Whether gaze-related behaviors signal sexual or relationship availability has yet to be explored and could be of interest to evolutionary researchers. Study 2 was designed primarily to examine correlations between the new scales and objectively measured gaze behavior via eye tracking, while also estimating test–retest reliability. However, several other hypotheses will be offered following the results of Study 1. The hypotheses for Study 1 were as follows:

#### H1

Using exploratory factor analysis (EFA), at least two moderately correlated factors were expected which measure pervasive body gaze and body gaze provocation tendencies in both women and men.

#### H2

Using confirmatory factor analysis (CFA), the factor scales suggested by the exploratory factor analysis were expected to yield acceptable model fit for both women and men.

#### H3

Pervasive body gaze scores were expected to correlate positively with the ISOS-P body gaze scale, rape myth acceptance attitudes, and pornography use in both women and men.

#### H4

Body gaze provocation scores were expected to correlate positively with own-body attitudes, perceived sexual objectification by others toward the self, appearance self-esteem, sensation seeking, pornography use, and enjoyment of sexualization (in women).

#### H5

While the analysis of relationship status was largely exploratory, we expected that pervasive body gaze and body gaze provocation scores might differ such that they would be higher for those identifying as single compared to those who are partnered. However, we also expected that such effects may not be consistent for women and men as a reflection of their distinct mating goals.

## Study 1

### Method

#### Participants

Self-reported heterosexual participants (*n* = 1113) were recruited from the university and surrounding community. Following exclusion of participants who did fully complete all the new items, 1021 cases were used for the factor analyses, 299 (29.3%) were men and 722 (70.7%) were women. Participants were aged from 18 to 71 years old (*M* = 29.31, SD = 10.00). There was no difference in age (*p* = .46) between women (*M* = 29.16, SD = 10.15) and men (*M* = 29.67, SD = 9.65). The female and male samples were each randomly divided into two approximate halves so that the EFA (*N*_women_ = 357; *N*_men_ = 157) and CFA (*N*_women_ = 365; *N*_men_ = 142) could be conducted on independent data for men and women separately.

### Measures

#### Item Generation for Pervasive Body Gaze and Body Gaze Provocation

A pool of 48 statements were generated by the first two authors to capture an endorsement of/tolerance of/enjoyment of/preference to engage in pervasive body gaze and body gaze provocation behaviors. Pervasive gaze items were guided by the notion that attempts to gaze upon the bodies of the opposite sex would often be effortful, uninhibited, contextually ignorant, and sometimes covert. Body gaze provocation items were guided by the notion that attempts to draw body gaze from the opposite sex would likewise often be effortful, uninhibited, contextually ignorant, and in some cases, planned (e.g., style of dress). An effort was made to have statements which described varied strategies (e.g., clothing, gesture) but were still passive in nature (could occur without a direct interaction between people). Most items were phrased such that men were asked to rate their gaze toward *women* and their attempts to attract gaze from *women*, and women were asked to rate their gaze toward *men* and their attempts to attract gaze from *men*. That is, participants were presented with slightly different items in order to orientate participants to specifically consider their gaze behavior toward the opposite sex. Therefore, this scale was designed for use with heterosexual participants. Participants were asked to rate the items on a 5-point Likert scale anchored with (1) *Strongly disagree*, (2) *Disagree*, (3) *Neither agree nor disagree*, (4) *Agree*, and (5) *Strongly agree*. Composite scores were to be created using the average of the final items sets.

#### Body Gaze (Gervais et al., 2018)

Body gaze (non-gender specific) was measured using the recently adapted 6-item subscale from the perpetrator version of the Interpersonal Sexual Objectification Scale (Kozee et al., 2007). This scale measured the frequency in which respondents recall staring, leering, or gazing at the body or body parts of others. Participants were asked to rate the items on a 5-point Likert scale anchored from 1 (*Never*) to 5 (*Almost always*)*.* Composite scores were created using the average of the item ratings. The internal consistency estimate in the current sample was .84. Note that, because this scale was published after our data collection commenced, it was later added to the survey battery and completed by a smaller subsample of participants (*N* = 153).

#### Attitudes Toward Appearance (Schaefer et al., 2015)

We used the two Internalization subscales (10 items) from the Sociocultural Attitudes Towards Appearance Questionnaire 4 (SATAQ-4) to measure two components of appearance. Specifically, participants were asked to rate their preference to be thin with low body fat as well as their preference to be muscular/athletic. Participants were asked to rate the items on a 5-point Likert scale anchored from 1 (*Definitely disagree*) to 5 (*Definitely agree*)*.* Composite scores were created using the average of the item ratings for each subscale. The internal consistency estimate in the current sample for thin/low fat was .81 and .89 for muscular/athletic.

#### Interpersonal Sexual Objectification (Davidson et al., 2013; Kozee et al., 2007)

The Interpersonal Sexual Objectification Scale (ISOS) captures the frequency that a respondent recalls experiencing being sexually objectified by others through body gaze, body comments, and unwanted sexual advances. We opted to use two scores from the ISOS that are validly comparable for women and men for assessing the frequency in which participants perceive being sexually objectified by others (non-gender specific). Specifically, and in line with suggestions by Gervais et al. (2018), we opted not to score the original body evaluation subscale as it has not shown consistent psychometric properties for both women and men. As such, we used the total mean score as well as the unwanted sexual advances subscore for measuring perceptions of being sexually objectified. Because the unwanted sexual advances scale includes items related to non-consensual touching and harassment, it is essentially a measure of sexual assault victimization, by definition in the USA and Australia (Australian Bureau of Statistics, 2018; Office on Violence Against Women, 2020). One item differed in its wording (chest/breasts) for men and women. Participants were asked to rate the items on a 5-point Likert scale anchored from 1 (*Never*) to 5 (*Almost always*). Composite scores were created using the average of the item ratings. The internal consistency estimate in the current sample for the unwanted advances subscale was .86/.84 (women/men) and 94/.90 (women/men) for the total mean score.

#### Appearance Self-Esteem (Heatherton & Polivy, 1991)

Appearance self-esteem was measured using a 6-item subscale from the State Self-Esteem Scale (SESS). The scale measures a respondents’ perception of their own appearance as represented by weight, attractiveness, and body satisfaction. Participants were asked to rate the items on a 5-point Likert scale anchored from 1 (*Not at all*) to 5 (*Extremely*)*.* Composite scores were created using the sum of the item ratings. The internal consistency estimate in the current sample was .88.

#### Sensation Seeking (Hoyle et al., 2002)

The Brief Sensation Seeking Scale (BSSS) captured individual differences in sensation seeking propensity. This 8-item self-report measure was adapted from Form V of the 40-item Sensation Seeking Scale. Those scoring highly on this measure are more likely to engage in risky behavior. Participants were asked to rate the items on a 5-point Likert scale anchored from 1 (*Strongly disagree*) to 5 (*Strongly agree*)*.* A composite score was created using the average of the item ratings. The internal consistency estimate in the current sample was .81.

#### Pornography Use

Participants were asked to report how many days in a week on average they used pornography in the last month. They were also asked to estimate (in minutes) how much time on average they spent using pornography on those days. Multiplying responses from these two questions yielded a weekly estimate of pornography use in minutes. Pornography was defined for participants as “any kind of material aimed at creating or enhancing your sexual feelings or thoughts at the same time. This material might include explicit exposure and/or descriptions of human genitals and/or clear and explicit human sexual acts such as vaginal intercourse, anal intercourse, oral sex, masturbation, bondage, sadomasochism, and/or rape.”

#### Sexual Assault Blame Attribution Attitudes

We selected three subscales from the Illinois Rape Myth Acceptance Scale (Payne et al., 1999) to measure the propensity to blame female victims (e.g., many women secretly desire to be raped) and exonerate male perpetrators (e.g., rape happens when a man's sex drive gets out of control) of sexual assault. Specifically, items from victim blame subscales “she asked for it” (SA) and “she wanted it” (WI), as well as a perpetrator exoneration subscale of “He didn’t mean to” (MT) were rated by participants from 1 (*Not at all agree*) to 7 (*Very much agree*). Composite scores were created using the average of the item ratings in each subscale. All subscales yielded acceptable internal consistency (SA-.88; WI-.85; MT-.80). In accordance with our ethics approval, participants were offered an opt-out for these items (25 participants).

#### Relationship Status

Participants were asked to select their relationship status from a range of options (e.g., long term, short term, married, de facto or marriage-like relationship, single), these were converted to a binary score to represent two categories (in a relationship or single). Note that we excluded people from relationship analyses who selected “other” for their status (*N* = 22).

#### Enjoyment of Sexualization (Liss et al., 2011)

Female participants completed an 8-item Enjoyment of Sexualization Scale (ESS) to determine the extent to which they enjoy “feeling sexy” and promoting their bodies to men for their attention. Participants were asked to rate the items on a 5-point Likert scale anchored from 1 (*Disagree strongly*) to 5 (*Agree strongly*)*.* A composite score was created using the average of the item ratings. The internal consistency estimate in the current sample was .90. As these items have only been validated for use in women, only data from female participants have been reported. This scale was also added to the data collection partway through (*N* = 134).

#### Body Shame and Surveillance (Moradi & Varnes, 2017)

Female participants completed two revised subscales (13 items) from the Objectified Body Consciousness Scale (OBCS; McKinley & Hyde, 1996), which evaluate the extent to which respondents engage in surveillance of their own body and the shame they experience in relation to their body. Participants were asked to rate the items on a 7-point Likert scale anchored from 1 (*Strongly disagree*) to 7 (*Strongly agree*). Composite scores were created using the average of the item ratings for each subscale. The internal consistency in the current sample for body shame was .84 and .84 for surveillance. As these items have only been validated for use in women, only data from female participants have been reported.

### Procedure

Flyers and digital advertisements for the survey battery were placed on university and community noticeboards, and the study was also included as part of an undergraduate psychology research credit scheme. Participants accessed the survey link directly on Web pages, used a QR code on print flyers, or emailed the lead author for the survey link. Upon accessing the survey, participants provided informed consent and then completed an initial block containing questions to capture demographics (e.g., age, gender, sexual orientation), relationship status, and pornography use. Following this initial block, all the other measures were presented in a random counterbalanced order to diminish any possible order effects. The median completion time was 27 min. Participants either received course credit or entry into a gift card prize draw on completion.

### Data Analysis

All the analyses described were performed separately for female and male participants. In one half of each gender sample, an EFA was conducted using maximum likelihood estimation and oblique rotation (direct oblimin) to examine the factor structure and determine item retention. Several criteria were used to guide the number of factors extracted on each iteration of the EFA. These included examination of a scree plot (Cattell, 1966; Floyd & Widaman, 1995), the Kaiser–Guttman rule (eigenvalues equal to or greater than 1) (Guttman, 1954; Kaiser, 1960), and parallel analysis (comparison of eigenvalues to those obtained from randomly generated data sets) (Horn, 1965). Prior to the EFA, items without at least one inter-item correlation of .3 or more were first excluded. Following each iteration of EFA, items with primary loadings of less than .3 were removed (Floyd & Widaman, 1995). Items with cross-loadings were also removed (i.e., a secondary factor loading of .3 or higher or a loading discrepancy of .2 or less) (Schaefer et al., 2015). Furthermore, factors loaded on by two or fewer items were omitted to ensure the final factors offered sufficient stability (i.e., 3 or more items per factor) (Floyd & Widaman, 1995). During the item removal process, we also aimed to keep items consistent for women and men, if achievable through minimal removal.

In the remaining half of each gender sample, a CFA was then conducted using maximum likelihood estimation to confirm the factor structure suggested by the EFA. As recommended by Schumacker and Lomax (2010), several model-fit indices were used to evaluate the model; these were the RMSEA, the SRMR, and CFI. A RMSEA of ≤ .08, a SRMR of ≤ .05, and a CFI ≥ .90 were interpreted to indicate an acceptable fit (Byrne, 2013; Hu & Bentler, 1999; Schumacker & Lomax, 2010).

Internal consistency for the resulting subscales was assessed using Cronbach’s alpha. Alpha values of .70 or higher were considered acceptable (Nunnally & Bernstein, 1994). Concurrent and convergent validity was assessed using Pearson correlations between the body gaze scales and measures of body attitudes, preferences, and behaviors (ISOS-P, ISOS, SATAQ-4, SESS), sensation seeking (BSSS), pornography use, and sexual assault attitudes (IRMAS). We used independent sample t tests to quantify relationship status differences in pervasive and body gaze provocation separately for women and men. All correlation coefficients were corrected (dis-attenuated) for imperfect reliability using the method described by Crocker and Algina (1986), which has since been validated using latent modeling (Fan, 2003). In line with recommendations for individual differences researchers derived from meta-analysis, we interpreted *r* values of .10, .20, and .30 as relatively small, typical, and relatively large, respectively (Gignac & Szodorai, 2016).

## Results and Discussion

### Exploratory Factor Analysis: Women

Prior to performing the EFA, two items were removed for possessing low inter-item correlations (< .30). The initial EFA yielded four eigenvalues above one with parallel analysis suggesting seven factors and the scree plot suggesting two factors. A 7-factor extraction was very unclear, so a four-factor extraction EFA was conducted. Following the removal of seven items with low primary or cross-loadings, four slightly clearer factors were obtained. The four items on the fourth factor were removed as this factor had one item with a relatively low primary loading (.33) and removal of this item left the factor with an eigenvalue less than one. A subsequent EFA with a three-factor solution was obtained but still lacked clarity. To assist the improvement in the solution, and in the interests of enhancing theoretical consistency, we reviewed the items carefully to remove 11 items with suboptimal wording or which were less directly linked to the acts of encouraging gaze toward the self or gazing at others. Following these exclusions, parallel analysis and scree inspection both produced a clearer two-factor solution, which was further improved by removing two cross-loading items. Finally, we opted to discard eight items which yielded factor loadings of less than < .50 which left 14 items across two subscales entitled Body Gaze Provocation (9 items) and Pervasive Body Gaze (5 items). The Body Gaze Provocation scale accounted for 32.3% of the variance and the Pervasive Body Gaze scale accounted for 21.2% of the variance.

### Exploratory Factor Analysis: Men

Prior to performing the EFA, two items were removed for possessing low inter-item correlations (< .30). The initial EFA yielded four eigenvalues above one with parallel analysis suggesting five factors and the scree plot suggesting four factors. A five-factor extraction was very unclear, and the only two items loading sufficiently on the fifth factor were also removed as this was not sufficient to form a subscale. A four-factor EFA was conducted, and, following the removal of nine items with low primary or cross-loadings, four slightly clearer factors were obtained. In line with the aim to find factors with equivalent items to women, the same four items on the fourth factor were removed. An EFA with a clearer three-factor solution was obtained. In the interests of enhancing consistency, we removed the same items (eight) as for women with suboptimal wording or which were less directly linked to the acts of encouraging gaze toward the self or gazing at others. Following these exclusions, parallel analysis and scree inspection both produced a clearer three-factor solution, which was further improved by removing two cross-loading items. Finally, we opted to discard three items which yielded factor loadings of less than < .50 which left 18 items across three factors. In additional to the two similar factors found for women, we found evidence for a third factor in men which is related to a tolerance or enjoyment of body gaze from others. The strongest two factors were entitled Body Gaze Provocation (8 items) and Pervasive Body Gaze (6 items), and the third factor was entitled Body Gaze Tolerance (4 items). The Body Gaze Provocation scale accounted for 25.73% of the variance, the Pervasive Body Gaze scale accounted for 19.12% of the variance, and the Body Gaze Tolerance scale accounted for 11.05% of the variance.

In line with the aims of the study, we examined the items suggested by the EFA for women and men to determine if further exclusions would allow consistent items in the Body Gaze Provocation and the Pervasive Body Gaze subscales for each gender. As there were only two unique items for each gender in these two scales, and to enhance the utility of the scales to allow meaningful comparisons between women and men, we removed two items from the provocation subscale for women and one item from each of these subscales for men. This left five items in the pervasive scale and seven items in the provocation scale. This minor adjustment did not adversely affect the variance accounted for when a two-factor solution was reanalyzed via EFA for women (53.5% increased to 54.3%) and men (52.9% increased to 55.3%). Following these adjustments, the common items (two-factor solutions) across women and men and their factor loadings and eigenvalues are provided in Table 1. These factors were moderately to highly correlated (*r*_men_ = .29, *r*_women_ = .55). The items and loadings for the three-factor solution in men have been supplied in the supplementary materials. These results are supportive of the hypothesis (H1) that two correlated equivalent factors would emerge to measure pervasive body gaze and body gaze provocation in women and men.

**Table 1:** Factor loadings and eigenvalues obtained using maximum likelihood estimation with direct oblimin rotation

Item	Factor loadings			
	Body gaze provocation		Pervasive body gaze	
	Women	Men	Women	Men
1. Even if my clothes are not revealing, I still try and draw attention to my body	**.87**	**.86**	− .12	.01
2. I make an effort to behave in a manner which attracts attention to my body	**.73**	**.70**	− .01	.01
3. If I notice an attractive man/woman looking at my body, I try to keep his/her attention there	**.71**	**.62**	.12	.14
4. No matter where I am, I typically wear revealing clothing	**.68**	**.77**	− .07	− .15
5. I intentionally position myself to give men/women a better view of my body	**.66**	**.77**	.14	.04
6. If I’m wearing revealing clothing, it is because I want to gain the attention of men/women	**.61**	**.54**	.11	.12
7. Sometimes I touch parts of my body to draw attention from men/women	**.58**	**.72**	.09	.00
8. Even if a man/woman’s clothing is not revealing, I still try to look at his/her body	− .05	− .07	**.85**	**.85**
9. No matter where I am, I typically find myself looking at the bodies of men/women	.01	.08	**.85**	**.84**
10. Once I notice an attractive man/woman’s body, I have trouble not looking at it	.03	.10	**.70**	**.66**
11. I intentionally position myself to get a better view of the bodies of men/women	.08	.18	**.67**	**.65**
12. I often look at the bodies of men/women when they are unaware that I am looking at them	.04	− .12	**.65**	**.82**
Eigenvalues	5.08	4.79	1.17	2.18
Cronbach’s Alpha	.88	.88	.87	.88

Factor loadings ≥ .50 in boldface.

### Confirmatory Factor Analysis: Women

The items identified for women were subject to maximum likelihood estimation confirmatory factor analysis in the independent sample of women. The two-factor model achieved acceptable, but fell below excellent, fit standards, χ^*2*^(53) = 186.44, *p* < .001, RMSEA = .08, SRMR = .05, and CFI = .93. A comparison of single-factor solution with the same items in women showed much poorer fit, χ^*2*^(53) = 342.16, *p* < .001, RMSEA = .12, SRMR = .07, and CFI = .84. The AIC values also indicated that the two-factor model (AIC = 9576) was superior to a single-factor model (AIC = 9765).

### Confirmatory Factor Analysis: Men

The items identified for men were subject to maximum likelihood estimation confirmatory factor analysis in the independent sample of men. The three-factor model for men achieved acceptable, but fell below excellent, fit standards χ^*2*^(101) = 165.05, *p* < .001, RMSEA = .07, SRMR = .06, and CFI = .95. A comparison of single-factor solution with the same items as the three-factor solution found for men showed much poorer fit, χ^*2*^(77) = 390.85, *p* < .001, RMSEA = .17, SRMR = .12, and CFI = .70. According to AIC values, the three-factor model demonstrated comparatively better fit (5671) to a single-factor alternative (6070). Using the same items as the two-factor solution found in women also achieved acceptable fit, χ^*2*^(53) = 91.17, *p* < .001, RMSEA = .07, SRMR = .05, and CFI = .96. A comparison of single-factor solution with the same items as the two-factor solution in both women and men showed much poorer fit, χ^*2*^(54) = 327.51, *p* < .001, RMSEA = .19, SRMR = .13, and CFI = .72. According to AIC values, the two-factor model (4202) demonstrated comparatively better fit to a single-factor alternative (4436). As such, the model-fit results suggest that an acceptable two-factor model exists with comparable items for women and men, and there is also an acceptable three-factor model for men only. As our remaining analyses focused primarily on contrasting gender differences in descriptive statistics and correlations, we have focused on the subscales derived from the two-factor models in women and men as these were best supported statistically and offered the most utility for researchers interested in gender comparisons. These results are supportive of the hypothesis (H2) that acceptable model fit would be achieved for both women and men.

### Internal Consistency, Concurrent, and Convergent Validity

The EFA and CFA samples for each gender were recombined to estimate descriptive statistics, internal consistencies, and Pearson correlations with convergent measures. These values have been reported separately for women and men in Table 2.

**Table 2:** Descriptive statistics, internal consistency estimates, and convergent validity correlations

	Body gaze provocation		Pervasive body gaze	
	Women	Men	Women	Men
Mean (SD)	1.59 (.60)	1.89 (.77)	2.11 (.81)	2.81 (.96)
Cronbach’s alpha	.86	.89	.86	.88
Correlations				
ISOS-P—body gaze	**.50**	**.31**	**.62**	**.86**
SATAC4—thin/low fat	.17	**.21**	.15	**.21**
SATAC4—muscle/athletic	.17	**.47**	**.23**	**.28**
ISOS—overall mean	.16	**.58**	.12	**.38**
ISOS—unwanted advances	.10	**.34**	.09	**.22**
Appearance self-esteem	− .01	.04	− .08	− .10
Sensation seeking	**.27**	**.31**	**.29**	**.22**
Pornography use	**.23**	**.39**	.19	.18
IRMAS—she asked for it	.11	**.26**	.10	**.24**
IRMAS—she wanted it	**.31**	**.30**	**.25**	**.28**
IRMAS—he didn’t mean to	.18	.15	**.20**	.13
Enjoyment of sexualization	**.68**	–	**.54**	–
OBCS—body shame	.18	–	.14	–
OBCS—body surveillance	**.22**	–	.14	–

*ISOS−P* Interpersonal Sexual Objectification Scale (Perpetration), *SATAQ4* Sociocultural Attitudes Toward Appearance Questionnaire, *IRMAS* Illinois Rape Myth Acceptance Scale, *OBCS* Objectified Body Consciousness Scale. Pornography use measured in minutes per week. Typical (.20) to relatively large (.30+) effect sizes in boldface. Correlations corrected for imperfect reliability (except for pornography use). Due to positive skew, correlations with pornography were estimated using Spearman’s Rho

As can be seen in Table 2, men scored higher than women for body gaze provocation and pervasive body gaze. When compared statistically (equal variances not assumed), both differences were significant (*p* < .001), but the effect size was larger for the pervasive body gaze scale (*d* = .81) than the body gaze provocation scale (*d* = .46). The alpha estimates were excellent for both subscales across genders.

Moreover, in Table 2, for both genders, there were relatively large positive correlations between the ISOS-P body gaze scale and the pervasive body gaze scale. In women, relatively large correlations were observed between both the body gaze subscales and enjoyment of sexualization. In men, relatively large positive correlations were observed between body gaze provocation and the SATAC (muscle/athletic) as well as the ISOS overall mean. Sensation seeking yielded typical to relatively large positive correlations with gaze provocation across women and men. Pornography use yielded typical to relatively large positive correlations with body gaze provocation across women and men. Victim-blaming IRMAS subscales (“she wanted it”; “she asked for it”) also yielded typical to relatively large positive correlations with both the provocation and pervasive subscales. Appearance self-esteem failed to correlate with either of the body gaze subscales in women or men.

**Fig. 1:**
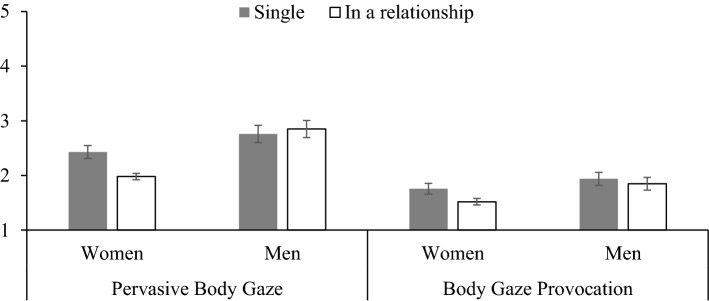
Mean pervasive and provocation scores reported separately for relationship status and participant gender. Error bars represent 95% confidence intervals

The pattern of correlations described above are largely supportive of the hypothesis (H3) that there would be positive associations in women and men between pervasive body gaze, the ISOS(P) body gaze scale, and rape myth attitudes. However, pornography use did not correlate with pervasive body gaze. Hypothesis 4 was only partially supported because there were no correlations between body gaze provocation and appearance self-esteem for women or men. However, body gaze provocation did positively correlate with body attitudes (muscle/athletic), perceptions of being sexually objectified (ISOS), pornography use, and sensation seeking for men. For women, there were similarly positive associations between body gaze provocation, pornography use, sensation seeking, body attitudes (surveillance), and enjoyment of sexualization. Finally, and as can be seen in Fig. 1, hypothesis 5 was supported only for women because single women were more likely to engage in pervasive body gaze (*p* < .001, *d* = .60) and body gaze provocation (*p* < .001, *d* = .40) behaviors than partnered women. When comparing single men and partnered men, there were no differences in their pervasive body gaze (*p* = .45, *d* =  − .09) and body gaze provocation behaviors (*p* = .30, *d* = .12). Further interpretation of these findings will be presented in the general discussion.

The next step in validating the new body gaze scales was to determine their associations with eye movements using eye tracking technology, as well as their test–retest reliability. While Study 2 was designed primarily to further validate the pervasive and provocation scales, it also offered an opportunity to examine general patterns of female and male gaze behavior while continuing to explore the associations between pornography use, rape myth acceptance attitudes with self-reported and objectively measured gaze behavior. Associations among these measures were anticipated to be valuable for continuing to develop a theoretical framework for sexual objectification. Note that we opted to use two objective gaze measures for estimating body gaze preferences, one was an absolute measure (time spent gazing at the body) and the other was a relative measure (time spent gazing at the body relative to the head or body-biased gaze). Both absolute and relative measures have been used in prior studies (e.g., Bareket et al., 2018; Gervais et al., 2013; Hollett et al., 2019; Karsay et al., 2018), and we hoped to shed further light on their comparative utility for correlating with sexually objectifying attitudes. As such, the following hypotheses were proposed for Study 2:

#### H1

Consistent with prior research (Lykins et al., 2008; Nummenmaa et al., 2012), men were expected to exhibit body-biased gaze (relative to the head) when presented with images of (a) partially clothed female subjects. In contrast, men were expected to exhibit head-biased gaze when presented with images of (b) fully clothed female or male subjects.

#### H2

Consistent with prior research (Lykins et al., 2008; Nummenmaa et al., 2012), women were expected to show body-biased gaze (relative to the head) when presented with (a) partially clothed women. In contrast, women were expected to exhibit head-biased gaze when presented with images of (b) fully clothed women or men.

#### H3

Absolute body gaze was expected to be greater for the partially clothed conditions overall, but this effect would be strongest for men when presented with partially clothed female subjects (when compared to women and fully clothed conditions). That is, an interaction effect was expected between dress type, participant gender, and subject gender.

#### H4

Pervasive body gaze behavior was expected to correlate positively with body-biased and absolute body gaze behavior in (a) men toward female subjects and in (b) women toward male subjects.

#### H5

Pervasive body gaze behavior was expected to correlate with rape myth acceptance attitudes and pornography use in both women and men.

#### H6

Absolute body gaze and body-biased gaze were expected to correlate with rape myth acceptance attitudes and pornography use in women and men.

#### H7

The provocative and pervasive gaze subscales were expected demonstrate acceptable test–retest reliability (> .50).

## Study 2

### Method

#### Participants

Self-reported heterosexual participants (*N* = 172) were recruited from the Study 1 sample. Of these participants, 167 (96 women, 71 men) were retained for gaze analyses and ranged in age from 18 to 61 years old (*M* = 29.83, SD = 9.39). There was no difference in age (*p* = .31) between men (*M* = 28.97, SD = 9.25) and women (*M* = 30.47, SD = 9.49).

### Materials

#### Eye-Tracking Imagery

**Fig. 2:**
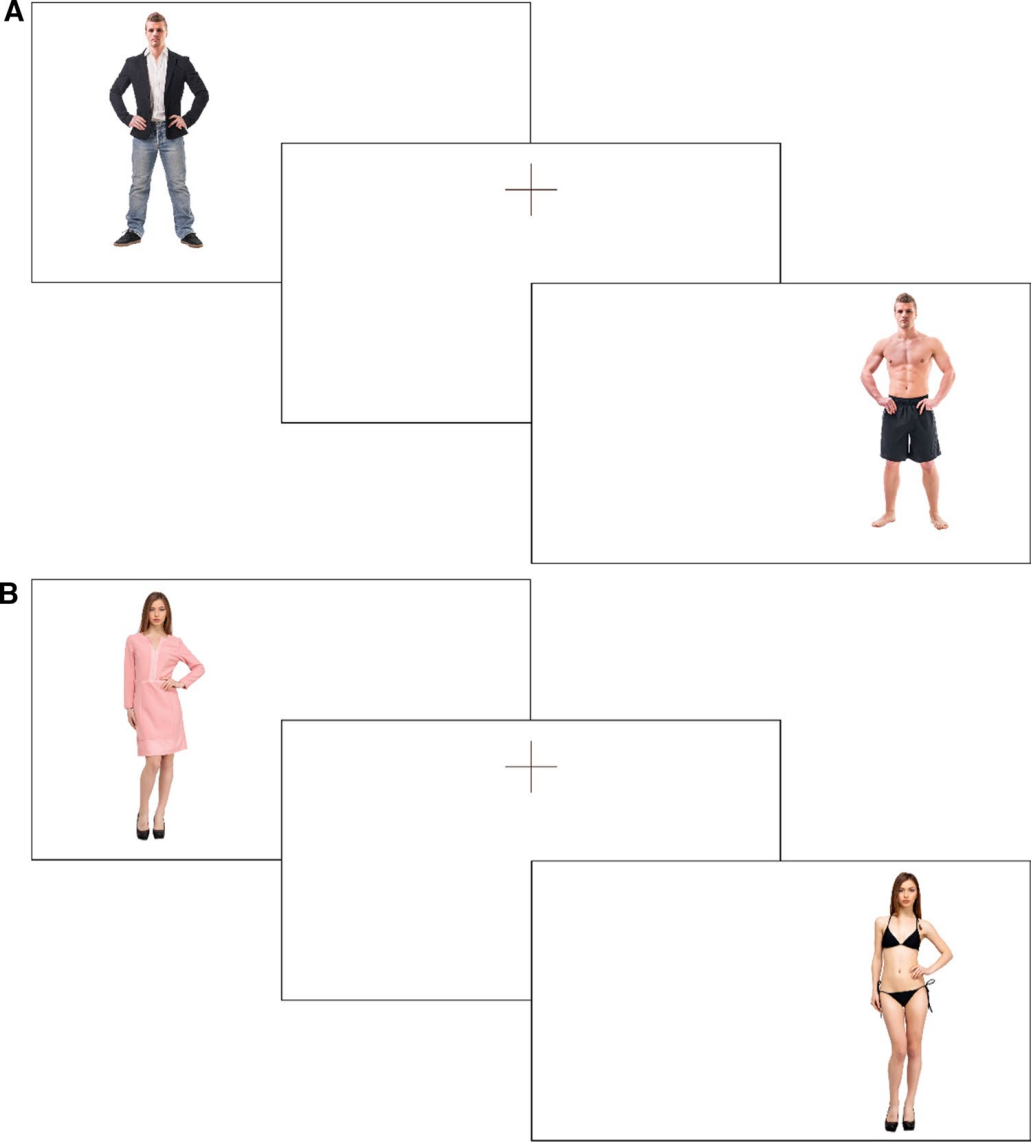
Examples of male (**A**) and female (**B**) subjects from fully clothed and partially clothed conditions, respectively

High-resolution studio photographs of female and male subjects were purchased from the Shutterstock library. (Image identifiers are provided in supplementary materials, shutterstock.com.) Subjects were only selected if they appeared in two suitably matched photographs (full-frontal body profile, visible facial features, similar posture, and an absence of props) but differed in their attire (partially and fully dressed). Specifically, the partially dressed photographs depicted subjects in swimwear, underwear, or shirtless (men), and the fully dressed photographs depicted subjects in casual or formal wear. Using these criteria, five female and five male subjects for each image condition were obtained, totaling 20 images displayed at 6.6 cm × 25 cm (visual angle (VA) of 5.90^○^ horizontally and 22.10^○^ vertically). Facial expressions were homogenized where necessary using Photoshop to ensure that each subject appeared comparable across their own respective partially and fully dressed images (e.g., see Fig. 2). Due to differing bodily proportions across characters, image sizes were standardized according to head dimensions (2.4 cm × 3.4 cm or 2.15^○^ of horizontal and 3.04^○^ of vertical VA). Two areas of interest (AOIs) were defined for each subject (head and body). The head AOI included the top of the head, hair, and face to the chin. The body AOI included the entire area below the chin.

#### Eye Tracking

Gaze data were sampled at 30 Hz using a screen-based Tobii eye tracker (X2-30) with up to .4° accuracy and .32° spatial resolution and mounted at the base of a 20-inch LCD screen. The female and male subjects were displayed using Tobii Pro Studio software on a Windows 7 operating system. Fixations were defined as consecutive gaze samples below a 30^○^/s velocity for a minimum duration of 100 ms using the Tobii Velocity Threshold Identification (I-VT) filter. In order to examine the degree of attentional processing to the head and body individually, a total fixation duration (in milliseconds) was calculated for each of these AOIs. For comparison with the previous literature, and to determine their relative value, we used two measurement variations of body gaze behavior. One was the total time spent gazing at the body (absolute body gaze), and the other was a *relative* score calculated by subtracting the time gazing at the head from the time gazing at the body to create a *body-biased* gaze score. That is, positive body bias scores indicated a preference for gazing at a subject’s body, whereas negative body bias scores indicated a preference for gazing at a subject’s head.

### Self-Report Measures

In addition to the self-report provocative and pervasive body gaze subscales identified via EFA and CFA in Study 1, we also analyzed pornography use and the three rape myth acceptance scales in Study 2 (see the Study 1 materials section).

### Procedure

Participants who completed Study 1 were invited to volunteer for Study 2 immediately following the completion of the online survey in Study 1. That is, only heterosexual participants were invited to complete the eye-tracking session. Participants who volunteered for Study 2 subsequently attended a laboratory at the university campus. The average time between participating in Study 1 and Study 2 was 19 days (SD = 18).

Prior to the eye-tracking task, participants were centrally positioned approximately 64 cm from the computer monitor and had their eye gaze calibrated. They were then informed that they would see several images of women and men, and were instructed to look at each image as they would normally look at a person. To offer a sense of purpose, participants were advised that they would be asked to provide some “ratings” about the images following the eye-tracking task. Each image was then presented in a random lateral location 12.70 cm (11.33^○^ horizontal VA) to the left or right of the center of the display for 4 seconds. The image sequence was randomized and then fixed with the order reversed for every other participant. Each image was presented twice (once on the left and once on the right side of the screen) with AOI fixations averaged across presentations. A break was offered halfway through the presentation. To orient gaze to the center of the display prior to each image exposure, a central fixation cross appeared at the location of the vertical boundary between the head and the body AOIs for 1 second. See Fig. 2 for an illustration of the stimuli presentation. Following the eye-tracking task, participants provided self-paced attractiveness ratings on a 6-point scale from 1 (*Very Unattractive*) to 6 (*Very Attractive*) of each character image and completed the pervasive body gaze and body gaze provocation items for the second time. Finally, participants were debriefed and remunerated with a gift card (AUD$20) or course credit. The entire session lasted approximately 20 min.

### Data Analysis

The current study employed a mixed factorial design which included two within-subjects factors (Dress Type [partially and fully clothed] and Subject Gender [male and female]) and one between-subjects factor (Participant Gender). The dependent variables for the gaze analyses were the two measures of objectively measured gaze behavior: absolute body gaze and body-biased gaze (described above). Cohen’s *d* was used to quantify the magnitude of all pairwise effects. To assess the expectations that both female and male participants would typically exhibit body-biased gaze for partially clothed female images and head-biased gaze for fully clothed images, a series of single sample *t* tests were conducted. This was done by comparing the body-biased gaze score to zero for each clothing and gender condition to determine whether women and men showed a body bias (positive score), a head bias (negative score), or a balanced gaze profile (score close to zero). ANOVAs were then used to detect main effects and interactions between the independent variables of Dress Type, Subject Gender, and Participant Gender on absolute body gaze behavior. That is, ANOVAs were used to test the hypothesis that body gaze would be strongest for men presented with female subjects, with absolute body gaze as the dependent variable. To test the hypothesis that self-reported and objectively measured gaze behavior were positively correlated, Pearson correlations were performed separately for men and women. To test the hypotheses that self-reported and objectively measured gaze behavior were positively correlated with rape myth acceptance and pornography use, we performed Pearson correlations between the two gaze measures (absolute and body-biased body gaze), the pervasive and provocation scales (Time 1 and Time 2), as well as rape myth acceptance scores, and pornography use, separately, for women and men. To evaluate test–retest reliability, we used a two-way random model of intraclass coefficient, in addition to reporting Pearson* r* between Time 1 (online survey) and Time 2 (laboratory session) measurements of provocative and pervasive gaze behavior. We adopted criteria for interpreting the intraclass coefficient as follows: .50 to .75 as moderate, .75 to .90 as good, and .90 or greater as excellent (Koo & Li, 2016).

## Results

### Data Screening

Prior to reporting descriptive statistics and testing the hypotheses, the gaze data were screened using gaze metrics provided by Tobii Studio. Using the average fixation durations spent not looking at any AOIs, we identified participants who were not engaging with the imagery consistently. Specifically, five participants were excluded for spending excessive time looking away from the stimuli (more than 3 *SD* units from the mean), leaving 167 cases for conducting gaze analyses. These exclusions assisted in reducing the impact of calibration problems, inconsistent attention, and data loss, which can be attributable to several, possibly systematic, factors (e.g., looking away from screen, eye physiology, obstructing the eye tracker with hands). Note that four cases could not be matched from Study 1 to Study 2 and were excluded from correlations between gaze measures and Study 1 self-report scores. Two additional cases did not complete the correct pervasive and provocative items for their gender in Study 1 and have been excluded from correlations involving those scores.

### Descriptive Statistics

Total fixation duration descriptive statistics for each area of interest and attractiveness ratings for each dress type and subject gender are presented in Table 3 (separated by participant gender). As can be seen in Table 3, at least numerically, the head of fully clothed male subjects attracted the most attention and the body of fully clothed male subjects attracted the least attention for women. However, for men, the body of partially clothed female subjects attracted the most attention and the head of partially clothed female subjects attracted the least attention.

**Table 3:** Descriptive statistics for total fixation and attractiveness by type of dress and Areas of Interest for male and female subjects

Type of dress	AOI	Men (*n* = 71)				Women (*n* = 96)				All participants (*n* = 167)			
		Fixation duration		Attractive		Fixation duration		Attractive		Fixation duration		Attractive	
		*M*	SD	*M*	SD	*M*	SD	*M*	SD	*M*	SD	*M*	SD
	(a) Male Subjects												
Fully clothed				3.39	.99			3.60	.92			3.51	.95
	Body	1331.77	797.64			1127.73	708.94			1214.48	752.43		
	Head	1465.44	954.95			1824.66	920.00			1671.93	949.03		
Partially clothed				3.51	1.07			3.66	.86			3.60	.96
	Body	1551.10	782.65			1370.73	826.64			1447.41	810.78		
	Head	1270.59	907.50			1610.76	1004.15			1466.14	976.11		
	(b) Female Subjects												
Fully clothed				5.07	.54			4.81	.64			4.92	.61
	Body	1645.31	759.02			1387.79	781.69			1497.28	780.34		
	Head	1235.94	847.93			1612.19	929.53			1452.23	912.39		
Partially clothed				5.08	.59			4.75	.69			4.89	.67
	Body	1841.13	816.20			1428.24	833.24			1603.78	848.80		
	Head	1048.14	773.84			1552.04	965.74			1337.81	921.25		

*AOI* area of interest. Fixation duration is in milliseconds. Attractive(ness) is rated on a 6-point scale

### Body-Biased Gaze Behavior

**Fig. 3:**
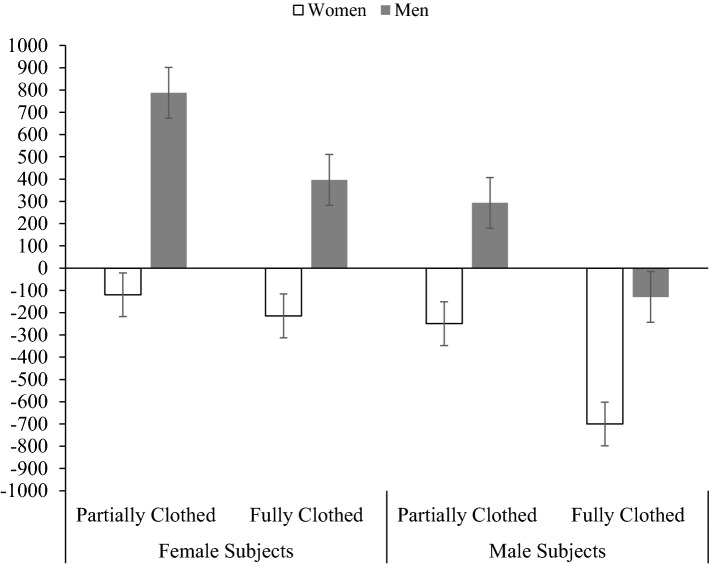
Mean difference scores between body and head fixation durations across dress conditions for men and women. Positive scores indicate a preference for the body, and negative scores indicate a preference for the head. Error bars indicate 95% confidence intervals

To illustrate relative gaze preferences (body vs. head) toward male and female subjects, a plot of the difference scores (i.e., calculated as average time spent fixating on the body subtracted from the average time spent fixating on the head) is shown in Fig. 3. Single sample *t* tests (comparing to 0) showed that men exhibited body-biased gaze when looking at fully clothed women (*p* = .027, *d* = .27), partially clothed women (*p* < .001, *d* = .54), but more balanced gaze profiles for fully (*p* = .501, *d* =  − .08) and partially (*p* = .115, *d* = .19) clothed men. This finding was partially supportive of the hypotheses (H1a and H1b) that men would show body-biased gaze behavior when looking at partially clothed female subjects, but not fully clothed female or male subjects. Single sample t tests (comparing to 0) showed that women exhibited head-biased gaze when looking at fully clothed men (*p* < .001, *d* =  − .45), but more balanced gaze profiles for fully clothed women (*p* = .202, *d* =  − .13), partially clothed women (*p* = .499, *d* =  − .07), and partially clothed men (*p* = .167, *d* =  − .14). This finding did not support the hypothesis (H2a) that women would show body-biased gaze behavior when looking at partially clothed female subjects. However, the hypothesis (H2b) that women would show head-biased gaze for fully clothed women or men was partially supported.

### Absolute Body Gaze Behavior

To determine which gender and image conditions had the strongest comparative body gaze effects, a 2 (Participant Gender: men; women) × 2 (Subject Gender: male; female) × 2 (Clothing Type: fully; partially) mixed-model ANOVA was performed. There was a main effect of Participant Gender, *F*(1, 165) = 5.3, *p* = .023, η_p_^2^ = .03, a main effect of Subject Gender, *F*(1, 165) = 61.20, *p* < .001, η_p_^2^ = .27, and a main effect of Clothing Type, *F*(1, 165) = 34.90, *p* < .001, η_p_^2^ = .17. However, there was a three-way interaction between dress type, participant gender, and subject gender, *F*(1, 165) = 4.90, *p* = .028, η_p_^2^ = .03. To decompose the three-way interaction, we conducted two follow-up 2 (Clothing Type: fully; partially) × 2 (Participant Gender: men; women) ANOVAs—one for female subjects and the other for male subjects.

For female subjects, there was a two-way interaction, *F*(1, 165) = 4.40, *p* = .037, η_p_^2^ = .03. Specifically, men showed significantly greater body gaze for the partially clothed condition (*M* = 1841.13, SD = 816.20) compared to the fully clothed condition, (*M* = 1645.31, SD = 759.02; *p* < .001, *d* =  − .25), whereas women showed no significant body gaze difference between the partially clothed (*M* = 1387.79, SD = 781.69) and fully clothed conditions (*M* = 1428.24, SD = 1272.24; *p* = .408, *d* = .05). Men also show greater body gaze (*M* = 1841.13, SD = 816.20) compared to women for the partially clothed condition (*M* = 1428.24, SD = 833.54; *p* = .002, *d* = .50) and fully clothed condition (*M*_men_ = 1645.31, SD_men_ = 759.02; *M*_women_ = 1387.79, SD_women_ = 781.69; *p* = .035, *d* = .33). Therefore, these results are consistent with the hypothesis (H3) that men would show greater body gaze toward female subjects for the partially clothed condition when compared to women and the fully clothed conditions, as indicated by the interaction.

For male subjects, there was a main effect of clothing type, *F*(1, 165) = 44.73, *p* < .001, η_p_^2^ = .21, but no main effect of participant gender, *F*(1, 165) = 2.70, *p* = .102, η_p_^2^ = .02, or an interaction, *F*(1, 165) = .117, *p* = .732, η_p_^2^ = .00. Specifically, participants, overall, showed greater body gaze for the partially clothed condition (*M* = 1447.41, SD = 810.78) compared to the fully clothed condition (*M* = 1214.48, SD = 752.43; *p* < .001, *d* = .30).

### Correlations Between Eye Movements and Self-Report

To examine associations between self-reported attitudes and gaze behavior, correlations between the two gaze metrics (absolute body gaze and body gaze relative to head gaze), pervasive and provocation scores, rape myth acceptance attitudes, and pornography use are reported in Table 4. For men, the pervasive body gaze scale yielded relatively large positive correlations (.33–.44) with absolute and relative body gaze but only for partially clothed female subjects. For women, pervasive body gaze was also positively correlated (.20–.24) with absolute and relative body gaze but only for partially clothed men, and only with small to typical strength. Therefore, the hypothesis (H4) that pervasive body gaze would correlated with objectively measured gaze behavior was only partially supported because body gaze and relative body gaze in women (toward men) and men (toward women) correlated with pervasive body gaze *only* for partially clothed subjects.

**Table 4:** Correlations for objective and self-report body gaze measures, Illinois Rape Myth Acceptance Scale, and pornography use, reported separately for men and women

Variables		Correlations										
		1	2	3	4	5	6	7	8	9	10	11
(a) Male participants												
1. Female body gaze (partial)		–										
2. Female body gaze (fully)		**.92**	–									
3. Female diff score (partial)		**1.00**	**.85**	–								
4. Female diff score (fully)		**.80**	**1.00**	**.91**	–							
5. Pervasive gaze (T1)		**.44**	.18	**.33**	.09	–						
6. Pervasive gaze (T2)		**.43**	.16	**.35**	.12	**.87**	–					
7. Gaze provocation (T1)		**.34**	**.22**	**.29**	**.21**	**.55**	**.47**	–				
8. Gaze provocation (T2)		**.44**	**.33**	**.39**	**.33**	**.57**	**.60**	**.80**	–			
9. IRMAS—she asked for it		**.31**	**.33**	**.33**	**.35**	.04	**.25**	**.35**	**.35**	–		
10. IRMAS—she wanted it		**.31**	**.29**	**.29**	**.27**	.17	**.27**	**.35**	**.35**	**.97**	–	
11. IRMAS—he didn’t mean to		.07	.11	.12	.19	.11	**.30**	**.43**	**.39**	**.74**	**.70**	–
12. Pornography use		.02	− .03	− .08	− .16	.13	.06	.01	− .01	.07	.11	− .10
(b) Female participants												
1. Male body gaze (partial)		–										
2. Male body gaze (fully)		**.97**	–									
3. Male diff score (partial)		**1.00**	**.96**	–								
4. Male diff score (fully)		**.89**	**1.00**	**.97**	–							
5. Pervasive gaze (T1)		**.23**	.14	.17	.08	–						
6. Pervasive gaze (T2)		**.24**	.13	**.20**	.08	**.72**	–					
7. Gaze provocation (T1)		**.34**	**.30**	**.34**	**.30**	**.44**	**.47**	–				
8. Gaze provocation (T2)		**.25**	.15	**.25**	.15	**.50**	**.76**	**.87**	–			
9. IRMAS—she asked for it		.16	.14	.19	.15	**.38**	**.37**	**.42**	**.39**	–		
10. IRMAS—she wanted it		**.37**	**.22**	**.38**	**.22**	**.30**	**.40**	**.66**	**.61**	**.42**	–	
11. IRMAS—he didn’t mean to		− .02	.03	.01	.08	.18	**.27**	.14	.15	**.62**	.03	–
12. Pornography use		.17	.06	.17	.06	.11	**.36**	**.28**	**.32**	.17	**.39**	− .05

*IRMAS* Illinois Rape Myth Acceptance Scale, *fully* fully clothed, *partially* partially clothed, *T1* Time 1, *T2* Time 2. Typical (.20) to relatively large (.30+) effect sizes in boldface

Correlations corrected for imperfect reliability (except pornography use). Due to positive skew, correlations with pornography were estimated using Spearman’s Rho

Pervasive body gaze in men was also significantly positively correlated with all three IRMAS subscales (.25–.30) but not with pornography use. Likewise, in women pervasive gaze was positively correlated with all of the rape myth acceptance subscales (.27–.40) and pornography use (.36). As such, and consistent with Study 1 and our Study 2 hypothesis (H5), pervasive body gaze correlated positively with rape myth attitudes and selectively with pornography use in men and women. In men, all the absolute and relative body gaze measures toward female subjects were positively correlated with the victim blame rape myth acceptance subscales (.29–.33). In women, only the absolute and relative body gaze toward partially clothed male subjects were positively correlated with one of the victim blame rape myth subscales (.22–.37). As such, these results are only partially supportive of our hypothesis (H6) because objectively measured body gaze was correlated with rape myth acceptance attitudes but not pornography use in men and women.

Test–retest reliability for both the pervasive and provocative subscales in men and for women was assessed using intraclass correlation coefficients. For men, the pervasive (.84) and provocative (.83) subscales yielded good test–retest reliability from Time 1 to Time 2. Similarly, for women, the pervasive (.76) and provocative (.82) subscales yielded good test–retest reliability from Time 1 to Time 2. Evidence of good test–retest reliability was consistent with our hypothesis (H7).

Regarding body gaze provocation, there was also a consistent pattern of relatively large positive correlations with rape myth acceptance attitudes, particularly for women (.39–.66). That is, women who engage in body gaze provocation are also more likely to endorse attitudes which attribute blame to female victims. Similarly, for men who engage in body gaze provocation, they were also more likely to endorse rape myth acceptance attitudes which attribute blame to female victims, as well as attitudes which exonerate perpetrators.

## General Discussion

The purpose of the present study was to develop a new measurement tool for the execution and provocation of sexually objectifying gaze. This objective was driven by the lack of available instruments in the literature and an assumption that recipients of body gaze behavior have some influence on, or a preference for, receiving body gaze. This recognizes that some recipients of body gaze might be intentionally seeking body gaze from others, or at least not deterring others from gazing at their bodies. We also attempted to embed pervasive elements (covertness, contextually ignorant, inhibition difficulties) into our scales to reflect the deleterious way sexually objectifying gaze has been described in the literature. Consistent with the aims of the study, we found evidence for two equivalent scales in men and women which estimate the propensity to engage in, and attempt to receive, body gaze. Both scales were validated through typical to large correlations with concurrent and convergent sexual objectification measures, good internal consistency, and test–retest reliability.

### Pervasive Body Gaze Behavior

The pervasive gaze behavior scale extends upon the body gaze scale developed by Gervais et al. (2018) by referring to a broader set of behaviors which involve a pervasive inclination to gaze at the bodies of the opposite sex. Consistent with research assuming that men are most likely to engage in sexually objectifying gaze patterns (Bareket et al., 2018; Gervais et al., 2018; Hollett et al., 2019), men scored significantly higher than women with respect to pervasive body gaze. We also found evidence that self-reported pervasive gaze correlated very strongly with the Gervais et al. (2018) body gaze scale for both women (.62) and men (.86). While these associations indicate considerable shared variability (38% and 74%, respectively), it also suggests that the new measure offers some distinction, particularly for women. That is, we can assume that women’s gaze behavior is less homogeneous than it is for men. Indeed, Gervais et al. (2018) reported in their follow-up questions that 51% of men report directing these types of behaviors toward “women only,” whereas only 26% of women reported directing these types of behaviors towards “men only.” This emphasizes the importance of more clearly differentiating sexual orientation and gender when measuring gaze, otherwise the motivations for performing the behavior are more ambiguous. A better understanding of the sexual orientation of the gazer and the gender of the recipient allows more confident conclusions regarding associations between gaze and gender attitudes.

Across both our studies, pervasive gaze behavior correlated with rape attitudes, specifically increased victim blame in women and men. While these associations might be more interpretable for men given the heterosexual nature of the sexual assault items (e.g., male perpetrator and female victims), it is interesting that they also exist for women. Specifically, men who pervasively gaze at women’s bodies may be more likely to assume that women invite or are tolerant of rough sexual conduct toward them. For women, their pervasive body gaze behavior toward men also correlated with victim-blaming attitudes. That is, women in our study who pervasively gaze at men’s bodies were more likely to attribute responsibility to women for their role in a sexual assault.

The possibility that pervasive body gaze is a marker of sexual or relationship interest was supported by the significantly higher pervasive body gaze scores in single women compared to partnered women. That is, pervasive body gaze in women toward men may represent a mate-seeking behavior. By contrast, in men, pervasive gaze behavior does not appear to be a marker for relationship status. That is, single men in our study were just as likely to engage in body gaze behavior as partnered men.

Importantly, self-reported pervasive body gaze correlated positively with objectively measured body gaze behavior in women and men but only for partially clothed subjects. This is somewhat unexpected as pervasive body gaze was assumed to persist under circumstances where recipient appearance does not necessarily encourage body gaze (e.g., fully clothed). While men showed body-biased gaze behavior when presented with fully clothed women during the eye-tracking task, this did not correspond with self-reported body gaze. While this can be explained in part by well-documented poor correspondence between self-report and behavioral methods (Dang et al., 2020), there are several other possibilities. For instance, the correspondence between body gaze behavior and self-report, particularly for men, might be greater for the partially clothed condition because it was more difficult to inhibit body gaze. Under conditions where the body is concealed, participants can inhibit these behaviors more easily, even if they are susceptible to exhibiting them in the real world.

We also suspect that the nature of the imagery may have restricted the associations between objectively and subjectively measured body gaze. Specifically, the subjects in the images exhibited perceived eye contact and were only viewed from a full-frontal angle which represents a fraction of real-world body gaze opportunities. That is, body gaze might be more likely when a subject is viewed from different angles (e.g., from behind) and when eye contact is absent. Research suggests that perceived eye contact can encourage mutual eye gaze (Senju & Hasegawa, 2005; Strick et al., 2008), thus facilitating head gazing behavior, rather than body gazing behavior. Our data confirm that, even if participants do self-report their real-world body gaze behavior accurately, it may still not correspond to laboratory gaze behavior because the visual stimuli will usually lack external validity. Researchers will soon be able to overcome these issues by employing virtual reality simulations to enhance fidelity and external validity. While eye tracking in virtual reality is still emerging (e.g., Clay et al., 2019; Meißner et al., 2019), we expect to soon see studies using this technology to explore sexual objectification.

### Body Gaze Provocation Behavior

The body gaze provocation scale was designed to estimate the extent to which people invite body gaze from others, which is argued to be fundamental for understanding the prevalence of sexually objectifying gaze in men and women. We found that women reported relatively low (*M* = 1.59) body gaze provocation behavior, and this was significantly lower than men (*M* = 1.89). As such, any argument claiming that women invite body gaze more so than men is not supported by our data. Interestingly, body gaze provocation in single women was significantly higher than partnered women, suggesting that single women might use body gaze provocation as a mate-seeking strategy. By contrast, relationship status did not differentiate men’s endorsement of body gaze provocation behaviors. This fits with research showing that women rely more heavily on self-sexualization as a mate-seeking self-promotion strategy, when compared to men (Bendixen & Kennair, 2015; Schmitt & Buss, 1996). Evolutionary scholars point out that self-promotion via appearance enhancement can include a range of strategies (e.g., cosmetics, clothing, hair styling/removal, dieting, and exercise), many of which directly affect bodily appearance and are similarly motived by mating goals (Davis & Arnocky, 2022). However, our body gaze provocation scale only captured a narrow range of these strategies (revealing clothing) and may not necessarily correspond to these broader appearance enhancement efforts which possibly better serve other goals, such as intrasexual competition (Darwin, 1871).

Our study has offered the first attempt to validate a body gaze provocation scale in women and men. For men, it correlated positively with all the body attitude measures, interpersonal objectification, pornography use, and sensation seeking. The finding that body gaze provocation behavior positively correlates with unwanted sexual advances in men is particularly notable, especially since this association was absent for women. That is, men who engage in more body gaze provocation are at greater perceived risk of receiving unwanted sexual advances. One limitation of the unwanted advances subscale is that it is not gender-specific, so it is unclear if these advances are from women or men. For women in our study, however, engaging in body gaze provocation does not appear put them at higher perceived risk of receiving unwanted sexual advances. This also implies that women using body gaze provocation as a strategy for securing sexual attention from men are unlikely to perceive subsequent sexual advances as “unwanted.” This pattern of results raises speculation regarding how sexual advances might be differently perceived by men and women and deserves more exploration. Women and men also showed positive correlations between body gaze provocation and pornography use across both studies, suggesting that exposure to pornography consumption might contribute to the adoption of exhibitory behaviors. This association was more consistent for women across the two studies. Because mainstream pornography tends to more often portray women as sexually provocative (Fritz & Paul, 2017; Klaassen & Peter, 2015), the modeling of provocative behavior by men in pornography, compared to women, may be less common, and thus less likely to encourage provocative behaviors in male consumers.

For both women and men, body gaze provocation was positively correlated with sexual assault attitudes. Most notably, in Study 2, women who reported engaging in higher body gaze provocation were more likely to endorse items on the “she wanted it” subscale (*rs* = .60–.66). These items largely refer to women enjoying or being tolerant of forced sex and rape. The strong positive associations (.38–.40) between body gaze provocation and the “she asked for it” subscale in women also suggest that women who engage in higher body gaze provocation recognize that body gaze provocation is perceived by men an invitation for sex. Findings which highlight links between provocation behaviors and elevated victim-blaming attitudes in women are helpful for explaining the underreporting of sexual assault (Kelly & Stermac, 2008; Taylor & Gassner, 2010), because some women may accept some responsibility for being victimized and are less likely to report an offense.

We also find evidence that sensation seeking positively correlated with body gaze provocation in women and men. This suggests that people with novelty-seeking traits are more likely to exhibit their bodies to others, and, given that sensation seeking positively correlates with number of sexual partners (Charnigo et al., 2013), such individuals may use this as a strategy for securing sexual partners. In women, the enjoyment of sexualization was also strongly positively associated with body gaze provocation, further suggesting a link between self-sexualization and exhibition of one’s body (Liss et al., 2011).

Regarding the overlap between self-objectification attitudes and body gaze provocation, we found more evidence of this in men than women. While body surveillance did positively correlate with body gaze provocation in women, body shame, desires to be thin or athletic, did not. By contrast, for men, the positive associations between body gaze provocation and desires to be thin and athletic suggest that body gaze provocation might be one manifestation of internalized self-objectification attitudes. Indeed, this is consistent with research showing that self-objectification attitudes in men correlate with desires to be muscular and their motivation to exercise for appearance (Oehlhof et al., 2009; Strelan & Hargreaves, 2005). Overall, the associations between body gaze provocation and body attitudes, rape attitudes, pornography use, and enjoyment of sexualization confirm that the body gaze provocation scale is an important feature of sexual objectification.

### Implications

Given that pervasive body gaze and body gaze provocation behaviors are both socially observable phenomena, the results of the current study raise several implications. Specifically, these behaviors might be useful social markers of maladaptive sexual attitudes and intentions for both women and men. Sexual objectification and its related mechanisms are helpful for explaining how people’s social and emotional well-being can be outweighed or neglected due to the value of their sexual features and functions, particularly in the context of sexual assault (Bernard et al., 2015; Loughnan et al., 2013). By showing that gaze correlates with sexual assault attitudes, we have offered further evidence that gaze behavior might serve as one marker for individuals who might be at risk of becoming sexual predators or victims. It is also important to recognize that, under some circumstances, receipt of body gaze may be sufficiently harmful on its own, with victimized women reporting increased benevolent sexism and self-objectification attitudes (Holland et al., 2017; Kozee et al., 2007; Sáez et al., 2016). Therefore, broadening our capacity to measure body gaze is important for furthering our understanding of the consequences of sexual objectification on these recipients.

A key theoretical implication raised by the current study is recognizing the importance of body gaze provocation as a component of sexual objectification. The evidence presented here suggests that provocation behavior has a theoretical value for furthering our understanding sexual objectification in women and men, particularly regarding self-objectification in men and the endorsement of sexual assault attitudes in both genders. Thus, we hope that our conceptualization and corresponding measurement tool will encourage future researchers to incorporate body gaze provocation into their theoretical and empirical investigations.

### Limitations and Future Directions

While our study makes a valuable contribution to the sexual objectification literature, we acknowledge there are several limitations which constrain our conclusions. In particular, our lengthy self-report survey likely introduced measurement error and we did not control for social desirability. We also note that it would have been desirable to capture more data on participants’ sexual activities (e.g., number of sexual partners, sexual risk taking, perpetration of sexual assault), including the full ISOS-P scale (which was published partway through our data collection), as well as mating efforts (e.g., Albert et al., 2021). Indeed, we recognize that there is an important agenda moving forward to examine associations between body gaze behaviors with adaptive and maladaptive mating efforts.

With respect to the eye-tracking stimuli, we also note several limitations. Firstly, by using studio images from a professional database meant that, while the quality of the imagery was high, the models were somewhat homogenous in their attractiveness, age, and ethnicity. While our participant samples were diverse in age, they were also largely ethnically homogenous (mostly White). This limits the generalizability of our data to gaze behavior of White participants toward relatively attractive White subjects. Given that the visual appeal and ethnicity of a subject are known moderators of sexually objectifying gaze (Anderson et al., 2018; Gervais et al., 2013; Hollett et al., 2019), we encourage future researchers to use more diverse samples and gaze subjects with a greater range of ethnicity, age, and attractiveness.

Secondly, the use of full-frontal still imagery restricts external validity because real humans are dynamic and are viewed from multiple angles. Finally, the perceived eye contact from the subjects toward the observer may have also influenced gaze behavior. We suspect the propensity to engage in pervasive body gaze might be enhanced when a subject is viewed from behind and/or not giving perceived eye contact. We suggest that these factors (viewing angle, eye contact) be tested experimentally to understand the conditions under which an individual might be most vulnerable to receiving body gaze. We also encourage future researchers to use experimental and/or longitudinal methods to determine what lifestyle factors might facilitate pervasive body gaze. For instance, we found some limited evidence in women that pornography use correlates with self-reported gaze but not objectively measured gaze. Given that pornography is perhaps the most prolific example of body-biased visual media (Fritz & Paul, 2017; Klaassen & Peter, 2015), it would be worthwhile conducting experiments to determine if it is capable of priming body gaze behavior in women and men.

Despite the limitations, our studies also possess several strengths. Firstly, we used a two-study design where multiple waves of data were collected. Secondly, we also used a combination of self-report and behavioral methods to estimate and validate gaze behavior. Our sample is also one of the largest eye-tracking studies on sexual objectification to date, and one of the few which has measured the gaze behavior of women and men toward both women and men. Finally, our eye-tracking stimuli, when compared to previous studies, was of high quality with respect to the image resolution and subject matching across partial and fully clothed conditions. Prior studies comparing gaze for sexualized and non-sexualized subjects had notably lower rigor in the consistency of their stimuli across conditions (e.g., Nummenmaa et al., 2012). We recognize, however, the challenge in securing high-quality and well-matched imagery when seeking to generalize beyond the laboratory.

### Conclusions

In our study, we have extended the understanding of body gaze by using self-report and behavioral measures and associating them with features of sexual objectification. We have developed new measurement tools and described methods for capturing sexually objectifying gaze behavior that may be useful to future researchers. Importantly, the new scales we have developed might afford some researchers without access to eye tracking to meaningfully contribute to scholarship on sexually objectifying gaze behavior. Our results offered notable associations which further inform the theoretical framework surrounding sexual objectification. Key conclusions are as follows.

Elevated pervasive gaze behavior in women toward men may be a marker that a woman is single, uses pornography, is more likely to blame female sexual assault victims, and assumes that women tolerate rough sexual conduct. Elevated body gaze provocation in women toward men may be a marker that a woman is single, uses pornography, is high in sensation seeking, enjoys being sexualized, is more likely to blame female sexual assault victims, and assumes that women tolerate rough sexual conduct.

Elevated pervasive body gaze in men toward women may be a marker that a man is more likely to blame female sexual assault victims, and assumes that women tolerate rough sexual conduct. Elevated body gaze provocation in men toward women may be a marker that a man self-objectifies, is high in sensation seeking, is more likely to blame female sexual assault victims, and assumes that women tolerate rough sexual conduct. A key finding was that men who engage in body gaze provocation toward women also reported being at a higher perceived risk of being sexually assaulted, but this was not the case for women who engage in body gaze provocation behavior.

## Supplementary Information

Below is the link to the electronic supplementary material.

Supplementary file1 (DOCX 15 kb)

## Data Availability

The data are available by request from the first author.
