# Supplementary material for: Body Gaze as a Marker of Sexual Objectification: A New Scale for Pervasive Gaze and Gaze Provocation Behaviors in Heterosexual Women and Men
Source: Arch Sex Behav. 2022 Mar 29;51(6):2759–80. doi: 10.1007/s10508-022-02290-y (PMC9363378; doi:10.1007/s10508-022-02290-y)
Supplement: Supplementary file 1 — Supplementary file1 (DOCX 15 kb) [file 10508_2022_2290_MOESM1_ESM.docx]

Image Identifiers

<https://www.shutterstock.com/>

| **Subject Gender** | **Condition** | **Shutterstock Identifier** |
| --- | --- | --- |
| Female 1 | Fully clothed | 497376931 |
|  | Partially clothed | 497378743 |
| Female 2 | Fully clothed | 334789574 |
|  | Partially clothed | 521977189 |
| Female 3 | Fully clothed | 434669359 |
|  | Partially clothed | 434665111 |
| Female 4 | Fully clothed | 500343154 |
|  | Partially clothed | 502713184 |
| Female 5 | Fully clothed | 367927208 |
|  | Partially clothed | 347977241 |
| Male 1 | Fully clothed | 305358041 |
|  | Partially clothed | 276450200 |
| Male 2 | Fully clothed | 71824282 |
|  | Partially clothed | 71890072 |
| Male 3 | Fully clothed | 145299559 |
|  | Partially clothed | 145298605 |
| Male 4 | Fully clothed | 293987849 |
|  | Partially clothed | 293987642 |
| Male 5 | Fully clothed | 155761805 |
|  | Partially clothed | 155499041 |
